# Enhancing Cushing’s disease diagnosis: exploring the impact of desmopressin on ACTH gradient during BIPSS

**DOI:** 10.3389/fendo.2023.1224001

**Published:** 2023-08-03

**Authors:** Tobias Skrebsky de Almeida, Ticiana da Costa Rodrigues, Fabíola Costenaro, Leandro Armani Scaffaro, Maurício Farenzena, Fernando Gastaldo, Mauro Antônio Czepielewski

**Affiliations:** ^1^ Postgraduate Program in Medical Sciences: Endocrinology, Universidade Federal do Rio Grande do Sul (UFRGS), Porto Alegre, Brazil; ^2^ Endocrinology Division, Hospital Moinhos de Vento, Porto Alegre, Brazil; ^3^ Endocrinology Division, Hospital de Clínicas de Porto Alegre, Universidade Federal do Rio Grande do Sul (UFRGS), Porto Alegre, Brazil; ^4^ Radiology Division, Hospital de Clínicas de Porto Alegre, Universidade Federal do Rio Grande do Sul (UFRGS), Porto Alegre, Brazil; ^5^ Department of Radiology, Faculty of Health Sciences, McMaster University, Hamilton, ON, Canada

**Keywords:** Cushing’s syndrome, Cushing’s disease, ectopic ACTH syndrome, bilateral inferior petrosal sinus sampling, ACTH, desmopressin

## Abstract

**Introduction:**

The differential diagnosis between Cushing’s disease (CD) and ectopic ACTH syndrome (EAS) is complex, and bilateral inferior petrosal sinus sampling (BIPSS) is considered the gold-standard test. However, BIPSS with corticotropin-releasing hormone (CRH) stimulation is rarely available.

**Objective:**

This retrospective cohort study aimed to assess the accuracy of the inferior petrosal sinus to peripheral ACTH gradient (IPS:P) before and after desmopressin stimulation for the differential diagnosis of ACTH-dependent Cushing’s syndrome (CS), applying different cutoff values.

**Methods:**

A total of 50 patients (48 with CD and 2 with EAS) who underwent BIPSS were included in this study. The sensitivity and specificity of IPS:P in BIPSS before and after desmopressin stimulation were evaluated. Various cutoff values for IPS:P were examined to determine their diagnostic accuracy.

**Results:**

Using the traditional IPS:P cutoff, the sensitivity was 85.1% before stimulation, 89.6% after stimulation, and a combined sensitivity of 91.7%. Applying cutoff values of IPS:P >1.4 before and >2.8 after stimulation, the sensitivity was 87.2% and 89.6%, respectively, with a combined sensitivity of 91.7%. Receiver operating characteristic (ROC) curve analysis determined optimal cutoff values of 1.2 before stimulation and 1.57 after stimulation, resulting in a sensitivity of 93.6% and 93.8%, respectively, with a combined sensitivity of 97.9%. Specificity remained at 100% throughout all analyses. Among the 43 patients who responded positively to stimulation, 42 (97.7%) did so within the first three minutes, and all 43 (100%) did so within the first five minutes. None of the assessed clinical variables predicted the ACTH response to stimulation in BIPSS with statistical significance.

**Discussion:**

ACTH stimulation with desmopressin during BIPSS improves the accuracy of IPS:P, making it a valuable tool for investigating ACTH-dependent Cushing’s syndrome. Considering the low risk of complications, we recommend the use of desmopressin stimulation during BIPSS for the differential diagnosis of ACTH-dependent CS.

## Introduction

Cushing Syndrome (CS) is a rare disease that results from chronic exposure to elevated cortisol levels. It can be caused by either endogenous or exogenous factors, and its incidence is estimated to be 0.7-3.2 cases per million per year (1, 2). The mortality rate for CS is elevated and may remain higher than the general population even after remission of hypercortisolism (3, 4). The causes of endogenous CS are traditionally classified into two categories: ACTH-dependent (about 80-85% of cases) and ACTH-independent (15-20% of cases) (5). The most common cause of ACTH-dependent CS (75-80% of cases) is Cushing Disease (CD), which is characterized by a corticotropic pituitary adenoma. The remaining cases (15-20%) of ACTH-dependent CS are caused by ectopic ACTH syndrome (EAS), which occurs when tumors of various sites, histological differentiation, and aggressiveness produce ACTH. There are also exceptionally rare cases (<1%) of ectopic CRH-producing tumors (5, 6).

CS diagnosis is a complex and challenging pathway due to the variable pattern of hormonal findings, the non-specificity of clinical presentation, particularly in mild hypercortisolism states (7), and the technical limitations of diagnostic tests. Once CS is confirmed, it should be differentiated between ACTH-dependent or -independent cases (8). ACTH levels <10 pg/ml suggest an adrenal cause; ACTH levels >20 pg/ml suggest ACTH-dependent causes; and levels between 10-20 pg/ml are considered indeterminate, requiring additional tests to establish the etiology (5, 8). When ACTH-dependency is confirmed, the next diagnostic step is the differentiation between CD and EAS. In this step, non-invasive tests are initially recommended, such as the CRH test (CRH-t), the 8 mg dexamethasone suppression test (DST-8 mg), and a pituitary magnetic resonance imaging (MRI) (5, 8). These tests, however, presents heterogenous results, depend on the availability of CRH, restricted in many countries including Brazil, and present low discriminatory power (9, 10). An alternative to CRH-t is the use of desmopressin, which stimulates ACTH release in most patients harboring ACTH-secreting pituitary adenomas. The use of this stimulus for the differential diagnosis of CD vs EAS is controversial, since studies have demonstrated that EAS patients may present ACTH elevation following desmopressin administration (11–14). The DST-8 mg is widely available; however it also presents limitation due to the variability of criteria used; furthermore, it has shown insufficient discriminatory capacity in some studies (15, 16). Pituitary MRI fails to detect adenomas in CD patients in about 30-50% of cases even with modern technology equipment (17); moreover, it may also generate false-positive results since pituitary incidentalomas are common in the population, including macroadenomas (18). In cases of conflicting non-invasive test results and unavailability of other methods, bilateral inferior petrosal sinus sampling (BIPSS) should be performed to detect a central-to-peripheral ACTH gradient that allows the localization of the ACTH production (5). Some authors and guidelines recommend performing BIPSS in all patients with pituitary lesions < 6 mm demonstrated on MRI (5, 8, 19), whereas others suggest BIPSS should routinely be performed, especially to guide surgical therapy of CD (20–23). Thus, the procedure is considered the gold-standard in the differential diagnosis of ACTH-dependent CS, preferentially performed with CRH or, less frequently, with desmopressin. The use of CRH is a limiting factor since it is unavailable in many countries. On the other hand, although used in some medical centers, desmopressin as a stimulus for BIPSS is still poorly debated and assessed in the literature, and its utility in this setting remains uncertain since studies validating it in different populations and in larger series are still lacking (8, 24–26). A recent study evaluating desmopressin in a large cohort of patients proposed new diagnostic criteria, questioning the need of stimulus with the new cut-offs (27). Thus, the aim of this study is to assess the role of central-to-peripheral ACTH gradient after stimulus with desmopressin during BIPSS for the differential diagnosis of ACTH-dependent CS in a cohort of patients followed-up in a referral center for CS in Brazil.

## Patients and methods

### Patients

Between 1998 and 2020, 107 patients with ACTH-dependent CS were retrospectively evaluated at the Neuroendocrinology clinic of a tertiary center in Southern Brazil for BIPSS under desmopressin stimulation during initial diagnostic evaluation or after recurrence. Of these, 58 patients underwent BIPSS with desmopressin, 50 of which for the initial diagnostic evaluation, 7 after recurrence and 1 after emergency adrenalectomy. Eight patients who underwent BIPSS were excluded for insufficient data regarding final etiologic diagnosis (lack of histopatological confirmation, lack of biochemical remission 6 months after surgery, or lack of remission after radiotherapy). Finally, 50 patients were included in the analysis. The present study was conducted in compliance with the principles laid down in the Declaration of Helsinki and was approved by the Hospital de Clínicas de Porto Alegre Ethics Committee.

### Diagnosis of CS and ACTH-dependency status

After exhaustive screening for exogenous glucocorticoid administration, CS diagnosis was based on the presence of at least two of the following conditions: cortisol after low-dose dexamethasone suppression test (either 1 mg overnight or 0.5 mg 6/6 hours for 48h) > 1.8 µg/dL (DST-1mg); 24-h urinary free cortisol (UFC) or late night salivary cortisol consistently elevated in at least two samples (8). Additionally, late night serum cortisol > 7.5 µg/dL (8) and a desmopressin test (DES-t) with a peak ACTH > 71.8 pg/mL or an increase in ACTH ≥ 37 pg/mL from baseline (28) were also considered suggestive of CS.

After clinical and biochemical diagnostic confirmation of CS, plasma ACTH measurement classified CS into ACTH-dependent (ACTH > 20 pg/dL) or ACTH-independent (ACTH < 10 pg/dL). Values between 10-20 pg/dL were considered indeterminate and new samples were obtained for correct classification (8).

Next, patients diagnosed with ACTH-dependent CS underwent pituitary MRI for the identification of an adenoma. Due to the unavailability of CRH-t, it was rarely performed. The DES-t for the differential diagnosis of CD and EAS was considered predictive of CD when the increase was > 20% in cortisol or >35% in ACTH after stimulus. In virtue of its low accuracy, DST-8 mg was only performed in a few cases. Patients with inconclusive or negative imaging, those with adenomas < 6 mm or those with adenomas > 6 mm but discordant non-invasive tests were submitted to BIPSS with sampling of ACTH at baseline and after desmopressin stimulus.

After investigation, patients with a suggestive diagnosis of CD underwent transsphenoidal surgery. Histological confirmation of a pituitary adenoma staining positive for ACTH was considered the gold-standard for diagnosis. Additionally, patients with inconclusive or absent histological specimen who exhibited clinical and biochemical remission 6 months after surgery or who remitted after pituitary radiotherapy were also considered diagnosed for CD. The EAS cases were confirmed based on surgical excision or biopsy of tumoral lesions confirming the presence of ACTH-staining neoplastic cells.

### Bilateral inferior petrosal sinus sampling

The procedure was performed in the presence of documented hypercortisolism, in an angiography room, under sedation with fentanyl and midazolam, and by a qualified professional in interventional radiology. Initially, bilateral common femoral venipuncture was performed, maintained with 6 French (F) introducers. Then, ascending catheterization of the superior vena cava and internal jugular veins was performed with a 5F vertebral catheter and hydrophilic guidewire, with final positioning of the catheter tip at the level of the inferior petrosal sinuses. Angiographic confirmation was performed after injection of 10 ml of diluted nonionic contrast under digital subtraction, demonstrating bilateral sinus and sellar region opacification. In situations of fine-caliber inferior petrosal sinuses, a coaxial microcatheter was used for a better distal reach of the required topography. Heparinization was not usually necessary in this technique, only sequential washing of the catheters was performed between the sampling times with saline solution with 2 ml of heparin for each 1000 ml of solution. Samples were collected after washing the catheters at baseline. Then, 10 µg of desmopressin was administered intravenously and samples were collected after one, three, five, and 15 minutes. In some cases, the sampling times were slightly different, but always with one sampling at baseline and at least 3 samplings after stimulation. All samples were collected in ice-cold tubes, kept on ice and then centrifuged in a refrigerated centrifuge and frozen at -8°C until ACTH measurement, which occurred immediately after the end of the procedure. After the samplings, the catheters and introducers were removed, followed by manual compression of the inguinal region at the puncture site for 10 minutes, until complete hemostasis. After compression, a compressive dressing was placed at the puncture site and the patients remained at bed rest without flexing the thigh for 6 h. Our routine protocol in performing the BIPSS did not include the concomitant measurement of prolactin as suggested in some previous studies in the literature.

### Hormone assays

Until April 2004, cortisol was measured using a commercially available radioimmunoassay (RIA) kit (Diagnostic Systems Laboratories, Webster, TX, USA). From May 2004 to March 2010, the method was modified to an electrochemiluminescence immunoassay (ECLIA) kit (Modular Analytics E 170; Roche, Mannheim, Germany). From March 2010 to February 2014, cortisol was measured by chemiluminescence immunoassay (ADVIA Centaur XP Immunoassay System, Tarrytown, NY, USA). From February 2014 to October 2019, the method was Competitive Electrochemiluminescence. (Roche e602 equipment line). From October 2019 until the end of the study, the method was Microparticle Chemiluminescent Immunoassay. (Abbott equipment line). ACTH measurements up to February 2000 were performed by commercially available RIA. From February 2000 to April 2015, the method was chemiluminescence with the Immulite 1000 equipment. From May 2015 to April 2018, the method was electrochemiluminescence with the Roche e602 equipment. From May 2018 to August 2019, the method was sandwich electrochemiluminescence using the Roche e602 equipment. From August 2019 until the end of the study, the method was chemiluminescent immunoassay in the Immulite 2000 equipment. These assay differences do not show a large variation from normal values and as samples collected from the same patient were always analyzed with the same assay, the calculations of different indexes of central versus peripheral samplings did not change as a result of the trials. Of the cases studied, ACTH was measured by RIA in 1 patient, by Immulite 1000 in 35 patients, by Roche e602 *via* electrochemiluminescence in 9 patients, by Roche e602 *via* sandwich electrochemiluminescence in 4 patients and by Immulite 2000 in 1 patient.

The basal ACTH and UFC values, therefore, are presented according to the percentage above the ULN according to each methodology used at each moment. For the calculation of the ACTH inferior petrosal sinus to peripheral gradient (IPS:P), however, absolute values were used since the ratios are calculated for the same patient using the same assay.

### Statistical analysis

The Kolmogorov-Smirnov test was used to assess the distribution of variables. Continuous variables with normal distribution are presented as mean ± standard deviation (SD). Continuous variables with asymmetric distribution are shown as median and interquartile range (IQR). Categorical variables were compared using Fischer’s exact test. The comparison of continuous variables was performed using the Mann-Whitney test. ROC curves were used to assess the ability of the IPS:P gradient to discriminate between CD and EAS, and the Youden index was used to define optimal cutoffs. Sensitivity and specificity were calculated for the different criteria analyzed. Statistical analyzes were performed using the SPSS 24.0 program (statistical package software, SPSS Incorporation, Chicago, IL, USA). Differences were considered significant when p<0.05.

## Results

Patient characteristics are shown in **Table 1**. During the study period, 50 patients with a confirmed diagnosis of ACTH-dependent CS whose etiology could be confirmed through histopathological or biochemical data (remission after 6 months of surgery or after radiotherapy) who had undergone the BIPSS were included. The mean age (SD) at diagnosis was 38.22 (15.56) years, 39 patients (78%) were female, and 48 patients had CD and 2 EAS.

**Table 1:** Characteristics of studied patients.

Sex, n (%)	
Female	39 (78)
Ethnicity, n(%)	
White Other	45 (90) 5 (10)
Age at diagnosis^a^, mean ± SD	38.22 ± 15.56
ACTH^b^ (xULN), median (IQR)	1.14 (0.78-1.90)
DST-1mg^c^ (µg/dL), man ± SD	21.56 ± 13.57
Midnight cortisol^d^ (µg/dL),median (IQR)	23 (18.43-33.72)
UFC (xULN)^a^, median (IQR)	3.22 (2.14-6.49)
DST-8mg^e^, % supression, median (IQR)	84.04 (76.08-90.67)
Adenoma size in cm, median^f^ (IQR)	0.60 (0.4-1.1)
Microadenoma, n (%)	23 (46)
Microadenoma <0.6cm, n (% of microadenomas)	15 (65.2)
Macroadenoma, n (%)	8 (16)
Negative imaging, n (%)	19 (38)
BIPSS indication, n (%)	
Initial workup	44 (88) 6 (12)
Workup after recurrence Final etiology, n (%)	
CD EAS	48 (96) 2 (4)
Diagnostic confirmation of CD, n (%)	
Histopathological 6-month remission post-op Post-RDT remission	41 (85.4) 5 (10.4) 2 (4.1)

SD, standard deviation; xULN, times above Upper limito f normal; IQR, interquartile range; UFC, 24h urinary free cortisol; BIPSS, bilateral inferior petrosal sinus sampling; CD, Cushing Disease; EAS, ectopic ACTH syndrome; RDT, radiotherapy.

^a^n=49; ^b^n=47; ^c^n=29; ^d^n=45; ^e^n=11; ^f^n=31.

In the imaging results, 23 (46%) were microadenomas, among which 15 were < 0.6cm (65.2% of microadenomas), 8 were macroadenomas (16%), and 19 had negative or inconclusive imaging (38%). One of the patients with EAS had an image suggestive of a 0.4 cm microadenoma on MRI. Regarding macroadenomas, the indication for BIPSS was proposed based on the following situations: 3 presented with a clinical picture of EAS, including 2 with systemic lesions suspicious for neoplasia, 3 presented imaging characteristics that were somewhat atypical for adenomas, 1 was associated with a brainstem vascular lesion and one was a recurrent disease with postsurgical alteration and residual lesion.

BIPSS was performed in 44 patients who had not yet undergone investigation or treatment and in 6 patients who had been previously treated for CD but had relapsed during follow-up. No complications were recorded in any of the cases submitted to BIPSS. There were no thromboembolism events related to the procedure.

At baseline (before stimulation), 49 patients were evaluated (1 patient with CD had samples collected, but his results were not properly recorded). The median IPS:P gradient at baseline was 6.62 (IQR 2.46-11.36) in patients with CD and 1.14 (IQR 1.10-1.14) in patients with EAS (p=0.01). Using the IPS:P>2 gradient criteria, 40 of 47 patients with CD were positive and none of the 2 patients with EAS were positive, resulting in 85.1% sensitivity (95% confidence interval (CI) 71.1-93.3%) and 100% specificity.

**Figure 1:**
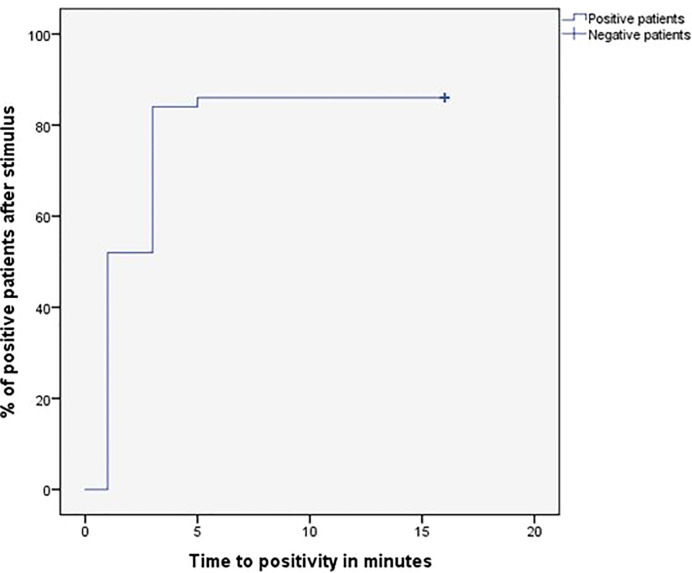
Time (minutes) until obtaining IPS:P gradient values of ACTH considered positive response of BIPSS after stimulation with desmopressin.

After stimulation with desmopressin, all 50 patients were evaluated. The median SPI:P gradient after stimulation was 29.46 (IQR 15.39-61.50) in patients with CD and 1.26 (min-max 1.25-1.28) in patients with EAS (p=0.01). In patients with EAS, the highest ACTH peak was 537 pg/mL (109.5% increase from baseline), while in patients with CD, the lowest increase from baseline was 19.48%. Using the IPS:P gradient criteria > 3, 43 of 48 patients with CD were positive, and none of the 2 patients with EAS were positive, resulting in 89.6% sensitivity (95%CI 76.5-96.1%) and 100% specificity. When evaluating patients who were positive at baseline and/or after stimulation in a combined manner, 44 of 48 CD patients were positive, whereas no EAS patients were positive. The overall sensitivity, therefore, was 91.7% (95%CI 79.1-97.3%), and the specificity was 100%. Of the 9 negative patients at baseline, 3 (33.33%) became positive after stimulation. Among the 43 patients who tested positive after the stimulus, 42 (97.7%) had already tested positive up to the third minute, and 100% of the patients were positive up to the fifth minute (**Figure 1**), totaling 86% of the total sample. Of the 3 patients whose stimulation was necessary, 2 had microadenomas and 1 had macroadenomas. In the two patients with EAS, the time of peak of ACTH was at 1 minute for patient 1 (31.1% increase from baseline) and at 3 minutes for patient 2 (109.5% increase from baseline).

When assessing only the 23 patients with microadenoma, 20 of 22 patients with CD were positive at baseline, and the patient with EAS and 0.4 cm microadenoma was negative, resulting in 90.9% sensitivity (95%CI 69.37-98.4%), while maintaining 100% specificity. After stimulation, all 22 patients with CD were positive and the only patient with EAS and microadenoma was negative, resulting in 100% sensitivity (95%CI 81.5-100%) while maintaining 100% specificity. When only microadenomas < 0.6 cm were evaluated, 12 of 14 CD patients were positive at baseline, and the patient with EAS and 0.4 cm microadenoma was negative, resulting in 85.7% sensitivity (95%CI 56.2-97.5), with 100% specificity. After stimulation, all 14 patients with CD were positive, and the patient with EAS and microadenoma was negative, resulting in a sensitivity of 100% (95%CI 73.2-100%) while maintaining 100% specificity. All eight patients with microadenomas >0.6cm were already positive at baseline and remained positive after stimulation (100% sensitivity and 100% specificity). Thus, only patients with microadenoma <0.6 cm improved sensitivity after stimulation. Among the 8 patients with macroadenoma, sensitivity was 75% at baseline and remained the same after stimulation. However, when assessed for need for stimulation, only one patient with macroadenoma benefited, but sensitivity did not increase because a patient who was positive at baseline became negative after stimulation. Assessing all patients with positive imaging on MRI (micro or macroadenomas, n = 31), 26 of 30 CD patients were positive at baseline, and the patient with EAS and microadenoma was negative, resulting in 86.7% sensitivity and 100% specificity. After stimulation, 28 of 30 CD patients were positive and the patient with EAS and microadenoma remained negative, resulting in 93.3% sensitivity and maintaining 100% specificity. The combined sensitivity (baseline or after stimulus) in this group of patients was 96.7%.

Among the 19 patients with negative imaging, 18 had baseline results and were evaluated. Baseline sensitivity was 82.4%. After stimulation, data from 19 patients were evaluated and resulted in a sensitivity of 83.3%. When the patients with negative imaging (n=19) and those with microadenomas <0.6 cm (n=15) were analyzed together, which represent the most difficult cases in clinical practice, we observed that the IPS:P gradient >2 at baseline resulted in sensitivity of 83.9% and 100% specificity. After stimulation, the IPS:P >3 gradient had a sensitivity of 90.6% while maintaining 100% specificity.

After assessing the traditionally proposed criteria, the analysis was performed using the criteria proposed by Chen et al. (27). Using the IPS:P gradient at baseline > 1.4, 41 of 47 CD patients were positive and none of the EAS patients were positive, resulting in 87.2% sensitivity (95%CI 73.5-94.7%) while maintaining 100% specificity. After stimulation, using the IPS:P>2.8 gradient criteria, 43 of 48 patients with CD were positive, resulting in 89.6% sensitivity (95%CI 76.5-96.1%), strictly the same as the traditional criteria maintaining 100% specificity. When evaluating patients who were positive at baseline and/or after stimulation, 44 of 48 patients with CD were positive, and no patient with EAS was positive, resulting in 91.7% overall sensitivity (95%CI 79.1-97.3%), the same as the traditional criteria. Finally, only 2 of 49 patients who were negative at baseline became positive after stimulation.

**Figure 2:**
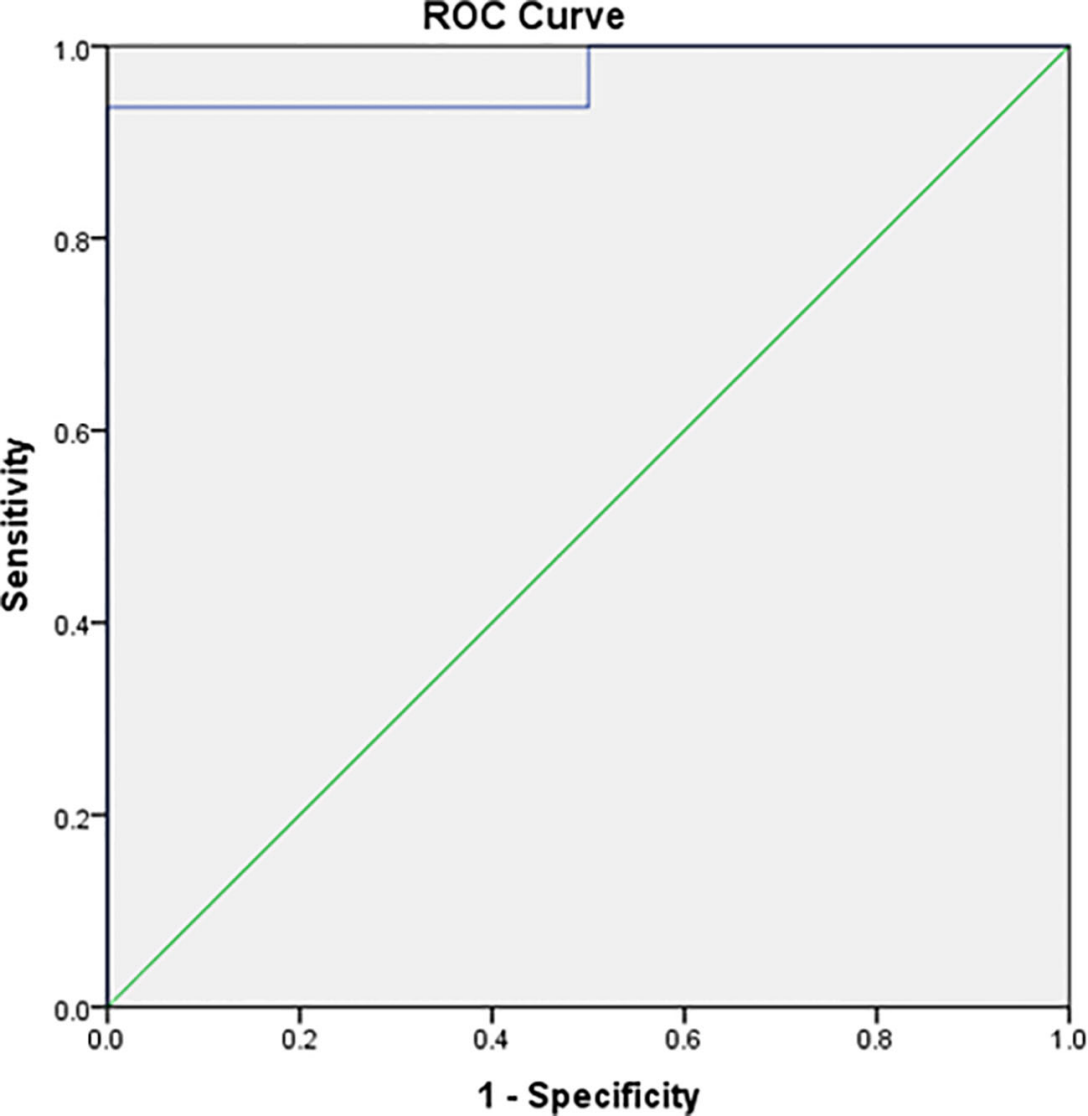
ROC curve of baseline IPS:P values in BIPSS in the investigation of ACTH-dependent CS.

**Figure 3:**
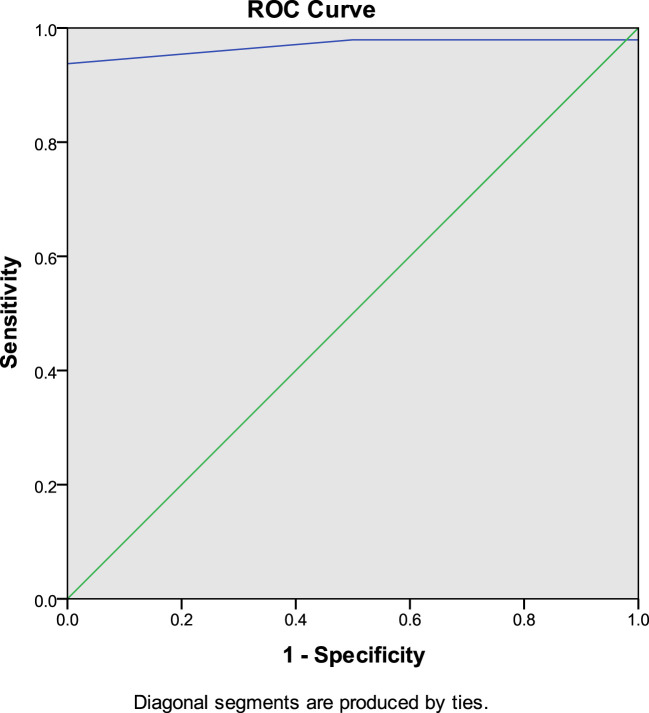
ROC curve of IPS:P values after stimulation with desmopressin in BIPSS in the investigation of ACTH-dependent CS.

To establish institution-specific cut-off points, a ROC curve was performed to assess the accuracy of the central/peripheral ACTH gradient in BIPSS in our cohort of patients. For the IPS:P gradient at baseline, the cut-off point with the highest accuracy was 1.2, whereas for the IPS:P gradient after stimulation, the cut-off point with the highest accuracy was 1.57 (**Figure 2**). Using these cut-off points, 44 of 47 CD patients were positive at baseline and no EAS patients were positive, resulting in 93.6% sensitivity (95%CI 81.4-98.3%), while maintaining 100% specificity. After stimulation, 45 of 48 CD patients were positive and no EAS patients were positive, resulting in 93.8% sensitivity (95%CI 81.8-98.4%), with 100% specificity (**Figure 3**). When evaluating patients who were positive at baseline and/or after stimulation, 47 of 48 CD patients were positive and no EAS patients were positive, resulting in an overall sensitivity of 97.9% (95%CI 87.5-99.9%) With 100% specificity. Finally, only 2 patients who were negative at baseline became positive after stimulation.

In the comparison between the traditional criterion and our study criterion, the baseline sensitivity changed from 85.1 to 93.6%. After stimulation, baseline sensitivity changed from 89.6 to 93.8%, respectively. A summary of the sensitivity results with the different diagnostic criteria is presented in **Table 2**.

**Table 2:** Sensitivity of BIPSS with traditional criteria and with present study criteria.

	Sensitivity	
	Traditional^a^	Our study^b^
IPS:P at baseline (n=49)	85.1%	93.6%
Microadenomas (n=23)	90.9%	95.2%
Microadenomas <0.6 cm (n=15)	85.7%	92.9%
Macroadenomas (n=8)	75%	87.5%
Negative imaging (n=18)	82.4%	94.1%
IPS:P after stimulus (n=50)	89.6%	93.8%
Microadenomas (n=23)	100%	100%
Microadenomas <0.6 cm (n=15)	100%	100%
Macroadenomas (n=8)	75%	75%
Negative imaging (n=19)	83.3%	94.4%
IPS:P at baseline and/or after stimulus (n=50)	91.7%	97.9%
Microadenomas (n=23)	100%	100%
Microadenomas <0.6 cm (n=15)	100%	100%
Macroadenomas (n=8)	87.5%	87.5%
Negative imaging (n=19)	83.3%	100%

a IPS:P >2 at baseline and IPS:P>3 after stimulus.

b IPS:P >2 at baseline and IPS:P>3 after stimulus.

Technical difficulties or anatomical variations were found in 6 patients undergoing BIPSS. Among the 43 cases with a positive IPS:P gradient, 3 had anatomical variations and 1 had some technical difficulty. Of the 5 cases in which the IPS:P gradient did not occur (false-negatives), 1 presented anatomical variation and 1 presented some technical difficulty during the test. Among the 6 patients who underwent BIPSS after recurrence, all had a final diagnosis of CD, and only 1 was negative on BIPSS.

Of the 50 patients evaluated, 43 had undergone DES-t as part of the diagnostic workup, of which 41 were later diagnosed with CD and 2 with EAS. Forty patients were considered responsive in DES-t, 38 patients with CD and 2 patients with EAS. Among the 40 responsive patients, 34 (85%) were also positive in BIPSS, all with a final diagnosis of CD. The 3 non-responsive patients in DES-t presented a positive response in BIPSS after desmopressin. Of the 6 patients who were positive in DES-t but negative in BIPSS, 2 were patients with EAS. Of the 4 patients with CD, 2 had normal petrosal sinus anatomy, 1 had a report of some anatomical variation, and 1 had a report of technical difficulties during BIPSS. Thus, DES-t was not able to predict response to desmopressin during BIPSS (p>0.9999). When comparing the ACTH values at baseline, 3, 5 and 10 minutes after stimulation in BIPSS, there was no significant difference between the group with positive versus negative DES-t, as well as no difference in the time to positivity between the groups, adenoma size, and number of patients with negative imaging. In addition, the clinical variables evaluated (ACTH, UFC, DST-1mg, baseline cortisol, adenoma size) were not able to significantly predict response to stimulus.

## Discussion

In this study, the use of BIPSS with ACTH measurements at baseline and after stimulation with desmopressin in the differential diagnosis of the ACTH-producing source in a sample of 50 patients with ACTH-dependent CS and inconclusive non-invasive tests resulted in 85.1% baseline sensitivity, increasing to 89.6% after stimulation, maintaining 100% specificity when applying traditional IPS:P≥2 criteria at baseline and ≥3 after stimulation (29). When combined, the baseline and/or stimulated sensitivity results were 91.7%. Results of meta-analyses that combined studies performed with CRH stimulation and desmopressin indicate that the sensitivity of BIPSS ranges from 86-97% and the specificity from 89-100% (27, 30). Published studies with desmopressin are generally small, with a variable number of cases of EAS, different indications for BIPSS, and variable diagnostic criteria. In a study with a sample of 56 patients with ACTH-dependent CS and negative imaging, using the criterion of IPS:P≥2 at baseline and IPS:P≥3 after stimulation with desmopressin, the combined sensitivity was 92.1% and 100% specificity, similar to the findings of the present study (25). Smaller studies that also used desmopressin stimulation found similar (26, 31–33) or slightly higher sensitivities (34, 35). Studies performed exclusively in pediatric patients were less uniform, with one of them reporting similar results to studies that included adults (36) and another study demonstrating lower sensitivity in adult population (37).

Our institution’s optimal cut-off points, determined by analyzing the ROC curve, were IPS:P≥1.2 at baseline and ≥1.57 after stimulation. This resulted in 93.6% baseline sensitivity (it was 85.1% with IPS:P≥2), and 93.8% after stimulation (was 89.6% with IPS:P≥3), and a combined sensitivity of 97.9% (it was 91.7%), maintaining specificity at 100%. Despite the increased sensitivity, these criteria should be used with caution, since the number of cases with EAS was small. The IPS:P gradient at baseline and after stimulation achieved in patients with EAS in some studies with desmopressin would exceed the cutoffs found by us (24, 25, 27), which would incorrectly classify these patients as CD. Before adopting the new values in our institution, therefore, more patients with EAS are necessary to validate these criteria. Also using the ROC curve, Castinetti et al. evaluated 43 patients with ACTH-dependent SC (36 DC and 7 EAS) and established the criteria of IPS:P>2 at baseline or after stimulation, obtaining a sensitivity of 86% at baseline and 97% after stimulation with desmopressin, not mentioning the combined sensitivity. The study, however, showed 85% specificity at baseline, given that a patient with EAS had a 3.33 gradient (24). In addition to applying the traditional criteria, Machado et al. also used ROC curve analysis to establish cut-off points, finding an IPS:P≥1.45 at baseline (88.2% sensitivity) and ≥ 2.04 after stimulation (92.2% sensitivity) as optimal, both with 100% specificity, although the authors did not recommend the use of these new values (25). The results of these studies using the ROC curve suggest that lower cutoff points, both at baseline and after stimulation, can improve sensitivity without compromising specificity. However, a study that performed a ROC curve in patients stimulated with CRH found an optimal 2.10 baseline cut-off, slightly higher than the traditional one of 2, although the post-stimulation cut-off point was 2.15, lower than the one usually used (38). A study with desmopressin, in turn, found values in the ROC curve of 1.76 at baseline, lower than the traditional one, but ≥3.9 after stimulation, higher than the gradient of three usually used, increasing baseline sensitivity but keeping the sensitivity after stimulation unchanged (32).

The largest published study evaluating BIPSS with desmopressin stimulation evaluated 226 patients with CD and 24 with EAS (27). Applying the IPS:P>2 criteria at baseline and >3 after stimulation, the sensitivity was 87.2 and 94.2%, respectively, while maintaining 100% specificity. The combined sensitivity was 96.5%. In this series, 3 cases of EAS reached gradients greater than 2 after stimulation, which suggests that cut-off points equal to or lower than this may decrease specificity. The authors also performed an ROC curve, determining the cutoff point of >1.4 at baseline and >2.8 after stimulation. In this analysis, the sensitivity at baseline was 94.7% and 96% after stimulation, resulting in a combined 97.8% sensitivity, higher than that found with the traditional criteria. According to the authors, with these cut-off points, only 7 patients benefited from the stimulus. After this publication, no other studies have tested these new cutoffs. Our study was the first, therefore, to assess the new values. In our series, using the cutoff point of >1.4 at baseline and >2.8 after stimulation, the sensitivity was 87.2 and 89.3%, respectively, and the combined sensitivity was 91.7%, thus slightly improving the sensitivity at baseline with little change after stimulation.

In an attempt to identify predictors of need for stimulation, Chen et al. found that patients requiring stimulation had adenomas < 0.6 cm or negative imaging. In addition, patients who required stimulation had lower IPS ACTH levels and did not lateralize. These data, however, are obtained only after performing the BIPSS, which makes their use in practice unfeasible (27). In our series, among patients with microadenomas, only those with lesions <0.6 cm benefited from the stimulus. Patients with negative imaging had a small increase in sensitivity. A patient with a macroadenoma also benefited from the stimulus, although the sensitivity of the cases with macroadenoma did not change, as a positive patient at baseline became negative after the stimulus. Despite current recommendations suggesting to perform BIPSS in patients with adenomas < 0.6 cm or with negative/inconclusive imaging results (8, 39), Chen et al. identified 2 patients with EAS and adenomas > 0.6 cm who would be misdiagnosed with CD if the 0.6 cm threshold were respected. Therefore, they suggest performing BIPSS in all patients with ACTH-dependent CS (27). Given the relevance of EAS cases in this study, a discussion about the current size criteria for indicating BIPSS should be undertaken.

Of our 50 patients, 43 (41 CD and 2 EAS) underwent DES-t prior to BIPSS, and 40 were considered responsive, including the two cases of EAS. Among the responders, 34 patients also responded to the stimulus during the BIPSS, all of them with CD. The 3 patients who did not respond to the peripheral stimulus were, however, positive in the BIPSS. The lack of correlation between the DES-t results and the BIPSS may be related to the different sampling intervals in the two exams (short intervals in the BIPSS and long intervals in the peripheral test). Considering that the majority (86%) of our patients performed both tests, it is possible to conclude that the DES-t did not help in the prediction of response to the central stimulus, which makes the use of peripheral test results debatable for this purpose. Of the BIPSS studies with desmopressin, only one described the results of DES-t, although it did not perform any specific analysis of the relationship with BIPSS (36). The study differs from ours, also, as it only evaluated pediatric patients.

Although BIPSS is still considered the gold standard in the differential diagnosis of ACTH-dependent CS, some authors have suggested that the procedure should be indicated only in cases in which t-CRH was negative (40, 41). Recent studies have evaluated non-invasive strategies combining t-CRH, DES-t, TSD-8mg, and imaging to reduce the need for BIPSS. Strategies that resulted in a positive predictive value of 100%, however, included t-CRH as part of the diagnostic process (42, 43), which makes adherence to this diagnostic modality inapplicable in many countries due to the unavailability of CRH. In one of these studies, the combination of TSD-8mg with DES-t, which would be possible in Brazil, was inferior to the combination of DES-t with t-CRH or t-CRH with TSD-8mg (43). The low number of patients undergoing TSD-8mg in our study did not allow the evaluation of this strategy. Although not recommended as a test in the differential diagnosis of the etiology of ACTH-dependent SC, DES-t seems promising as a marker of long-term postoperative outcome and as an early marker of recurrence (44), which encourages further studies in these circumstances.

Despite there have been reports of thromboembolic events related to BIPSS that occurred heparin (45, 46), it is a very rare complication. The administration of desmopressin, which increases coagulation factor VIII and von Willebrand factor (47), has raised concerns about the potential for increased incidence of thromboembolic events during BIPSS. This is due to the fact that desmopressin is associated with the hypercoagulable state of CS (48) and may also interfere with VIII and von Willebrand factors. The study by Chen et al, the largest published with desmopressin to date, did not record any case of thromboembolism, even without routine anticoagulation during the procedure (27). In our study, performed without routine anticoagulation, there were also no thromboembolic events. The only desmopressin BIPSS study that recorded thromboembolic events routinely used heparin during the procedure (25). Thromboembolic events, therefore, do not appear to be an additional concern when using desmopressin, with or without the use of heparin during the procedure. The decision regarding the use or not of anticoagulants during BIPSS should be a decision of each institution and based on the usual anticoagulation recommendations.

In our study, we did not perform the concomitant dosage of prolactin in samples collected from the inferior petrosal sinuses, a procedure that potentially reduces false negatives, as advised by some authors based on studies with CRH (49–51) and a study with desmopressin (31). These findings, however, were not confirmed by all groups, both with CRH (52) and with desmopressin (32), and their applicability depends on further studies to define its role.

In our study, a total of 3 patients who were negative at baseline benefited from the stimulus, As they became positive, 2 of them with microadenomas and one with macroadenoma. The study by Chen et al. questions the use of routine stimulation in all patients to reduce the risks and the duration of the procedure, potentially reducing complications. The authors argue that, when using the IPS:P>1.4 criterion at baseline, the sensitivity was high enough to classify most patients, with the exception of 7 patients with adenoma <0.6 cm who needed stimulation (27). Our study, however, would have misclassified a case with CD and macroadenoma as EAS if the stimulus had not been performed. The assessment of the need for stimulation in cases of CS with macroadenoma is limited since most studies performed the BIPSS only in patients with lesions < 0.6 cm or negative imaging, preventing a more comprehensive assessment.

Considering that BIPSS is currently still the gold standard in the differential diagnosis of ACTH-dependent CS, even small gains in sensitivity should be considered important since incorrect classification of patients can lead to inappropriate treatments and potentially fatal delays in the resolution of hypercortisolism. Considering that BIPSS is generally well tolerated and the rate of serious complications is low (53), other strategies to reduce the risks of the procedure that do not involve avoiding the stimulus seem necessary. In this context, it is important to evaluate the time interval between the infusion of the secretagogue and the positive test result. In our study, 97.7% of the patients who tested positive after stimulation were already positive in the third minute and 100% of the patients were positive until the fifth minute, demonstrating that there seems to be no benefit in prolonging the test beyond this period. All of the few studies on BIPSS with desmopressin have directly or indirectly reported a similar time to positivity and for peak ACTH (i.e., positive up to 3-5 minutes) (26, 33, 35, 37). Stimulating patients for a maximum time of 5 minutes considerably reduces the procedure time without neglecting the sensitivity gain resulting from the stimulation and may, therefore, be a strategy to potentially reduce the risk of complications.

Our study evaluated a sample of patients whose BIPSS indication was more comprehensive since the unavailability of t-CRH and the low accuracy of TSD-8mg limited the use of non-invasive tests. The wide heterogeneity existing in the BIPSS studies regarding the characteristics of the evaluated patients (primary diagnosis or recurrence), the BIPSS technique (sampling times, anticoagulant use, material used, laboratory assays, cut-off points, type of secretagogue) makes direct comparisons difficult. Conducting multicenter prospective studies with a greater sample of EAS patients is necessary to improve our understanding of the best cut-off points and procedure duration.

The present study has some limitations, as expected in the complexity of CS investigation. Our main limitation is that the low prevalence of EAS that underwent BIPSS, resulting from the rarity of this condition, may explain the high specificity when applying the cutoff points indicated by the ROC curve, and the application of these new gradients of IPS:P depends on validation in larger samples of EAS. Lower specificity may result from poor responsiveness to the secretagogue (desmopressin or CRH), cyclic CS during periods of normal cortisol secretion or due to anomalous venous drainage (54). Retrospective data collection and analysis prevented access to complete information for all patients. There were differences over time in terms of sampling times, although at least 3 different samplings were always performed throughout the study period. We highlight that, in this study, we did not discuss the data regarding the eventual lateralization of the basal ACTH values and after stimulation with desmopressin to guide the location of the pituitary adenoma in the transsphenoidal surgery. This utility of the BIPSS has been less and less recommended in the literature due to the imprecision of the results, especially due to the existence of venous communications between the cavernous sinuses and the instability and intensity of blood aspiration for sample collection.

In conclusion, in BIPSS with ACTH dosage, the use of stimulation with desmopressin increases the sensitivity of the test from 85.1% to 89.6%, reaching 100% in the sub-analysis of microadenomas. In spite of being small, this increase is useful in the investigation of ACTH-dependent CS, a clinical situation in which gains in diagnostic sensitivity are very important. Additionally, considering the low risk of complications and the possibility to interrupt the test within 5 minutes, as demonstrated in our study, our data recommend the use of stimulation with desmopressin in the BIPSS in the differential diagnosis of ACTH-dependent CS.

## Data availability statement

The raw data supporting the conclusions of this article will be made available by the authors, without undue reservation.

## Ethics statement

The studies involving human participants were reviewed and approved by Hospital de Clínicas de Porto Alegre Ethics Committee. Written informed consent to participate in this study was provided by the participants’ legal guardian/next of kin.

## Author contributions

TA, TR and MC conceived the study and designed the research. TA conducted the data collection and database management. TA performed the data analysis. LS, MF and FG performed the BIPSS procedures. TA, TR, FC and MC contributed to the interpretation of the results. TA and MC drafted the manuscript. FC critically revised the manuscript. All authors read and approved the final version of the manuscript. All authors contributed to the article and approved the submitted version.
